# Arthroscopic Management of Melorheostosis-Induced Ankle Deformity: A Case Report

**DOI:** 10.7759/cureus.68747

**Published:** 2024-09-05

**Authors:** Che-Yu Lin, Kai-Chiang Yang, Kuang-Ting Yeh, Meng-Chun Tsai, Chen-Chie Wang

**Affiliations:** 1 Orthopedic Surgery, Taipei Tzu Chi Hospital, New Taipei City, TWN; 2 School of Dental Technology, College of Oral Medicine, Taipei Medical University, Taipei, TWN; 3 Orthopedic Surgery, Hualien Tzu Chi Hospital, Hualien, TWN; 4 Orthopedics, School of Medicine, Tzu Chi University, Hualien, TWN; 5 Orthopedic Surgery, Buddhist Tzu Chi Medical Foundation, Taipei Tzu Chi Hospital, New Taipei City, TWN

**Keywords:** achilles tendon lengthening, arthroscopic management, candle-like flowing wax, melorheostosis, osteotectomy

## Abstract

Melorheostosis is a rare congenital sclerosing bone dysplasia characterized by a distinctive “candle-like flowing wax” radiological appearance. This study presents a case of an 80-year-old male who presented with a decade-long history of left ankle pain and rigid equinus deformity, which substantially impaired his mobility and activities of daily living. Conservative interventions, including physical therapy and custom orthotics, failed to alleviate his symptoms. Subsequent arthroscopic osteotectomy and Achilles tendon lengthening engendered substantial postoperative improvements in pain relief, range of motion, and gait stability, enabling him to resume previous activities such as cycling. This study highlights the potential of minimally invasive surgical techniques in managing melorheostosis-related deformities to optimize patient outcomes and quality of life. Nevertheless, long-term follow-up is essential for assessing the risk of recurrent ankle deformity and the potential requirement for revision surgery.

## Introduction

Melorheostosis - also known as candle bone disease, melting wax syndrome, or Leri disease - is a rare, congenital benign sclerosing bone dysplasia with a prevalence of 0.9 cases per million population [1]. First described by Leri and Joanny in 1922 [2], the term “melorheostosis” is derived from the Greek words “melos” (limb), “rhein” (flowing), and “ostosis” (bone formation), reflecting the characteristic “candle-like flowing wax” appearance on radiographic images of long bone diaphyses [3].

Melorheostosis predominantly affects the diaphysis of the lower limbs rather than the axial skeleton [3]. It is often associated with a restricted range of motion, osteoarthritis changes in adjacent joints, and potential entrapment of peripheral nerves or blood vessels, leading to considerable pain [4].

Debulking surgery to restore the range of motion and alleviate pain is the primary therapeutic approach for melorheostosis-related deformities [5]. Osteotectomy is a commonly employed symptom relief technique, and surgical decisions are dependent on patient age and lesion location. Herein, we reported a case of melorheostosis involving the ankle joint and treated with surgical intervention.

## Case presentation

**Figure 1:**
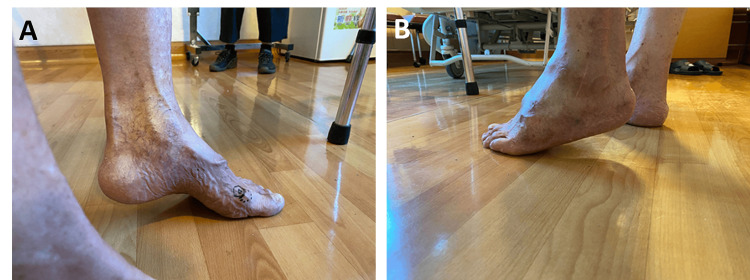
Preoperative appearance of the left ankle. The images show (A) medial view and (B) lateral view. Rigid equinus deformity can be observed.

An 80-year-old male presented to our orthopedic outpatient department in 2023 with chief concerns of left ankle pain (visual analog scale {VAS} score: 4) and limited dorsiflexion associated with a rigid equinus deformity (range of motion {ROM}: 20°-30° plantarflexion) and impingement symptoms that persisted for more than 10 years (Figures 1A, 1B). He was unable to achieve a plantigrade foot position when walking and required a heel lift insole to improve his gait pattern. The fixed equinus deformity precluded the patient’s participation in exercises that he previously enjoyed, such as mountain climbing and cycling. Pain, decreased stability during the stance phase of gait due to forefoot weight-bearing, and subsequent impairment of walking ability and activities of living (ADLs) constituted the primary clinical manifestations. The patient’s preoperatively American Orthopaedic Foot and Ankle Society (AOFAS) Ankle-Hindfoot score was 57/100.

His surgical history included an open left ankle cheilectomy and Achilles tendon lengthening (ATL) performed 12 years before (2011) at the National Taiwan University Hospital. Physical examination revealed a left ankle equinus deformity (Figures 1A, 1B). It also revealed hindfoot valgus, midfoot abduction, forefoot abduction, and medial longitudinal arch loss. Multiple irregular bony tubercles and spurs were palpable on the medial and lateral malleoli, talus, and midfoot, and these engendered impingement within the affected joints. Hyperpigmentation was observed on the left anterior lower leg and ankle.

**Figure 2:**
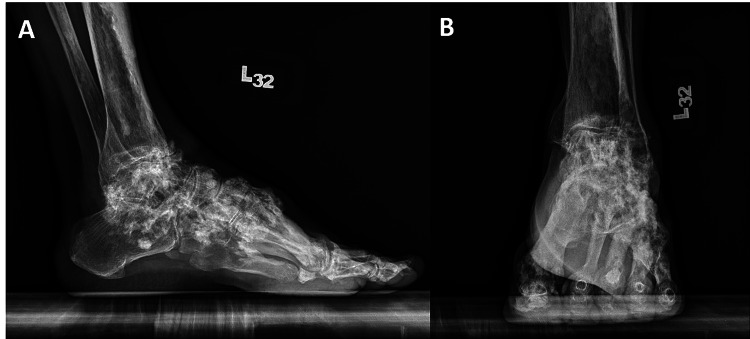
Preoperative weight-bearing radiographs of the left ankle. The images show (A) lateral view and (B) anteroposterior view. A classic “candle-like flowing wax” appearance can be observed, consistent with melorheostosis.

**Figure 3:**
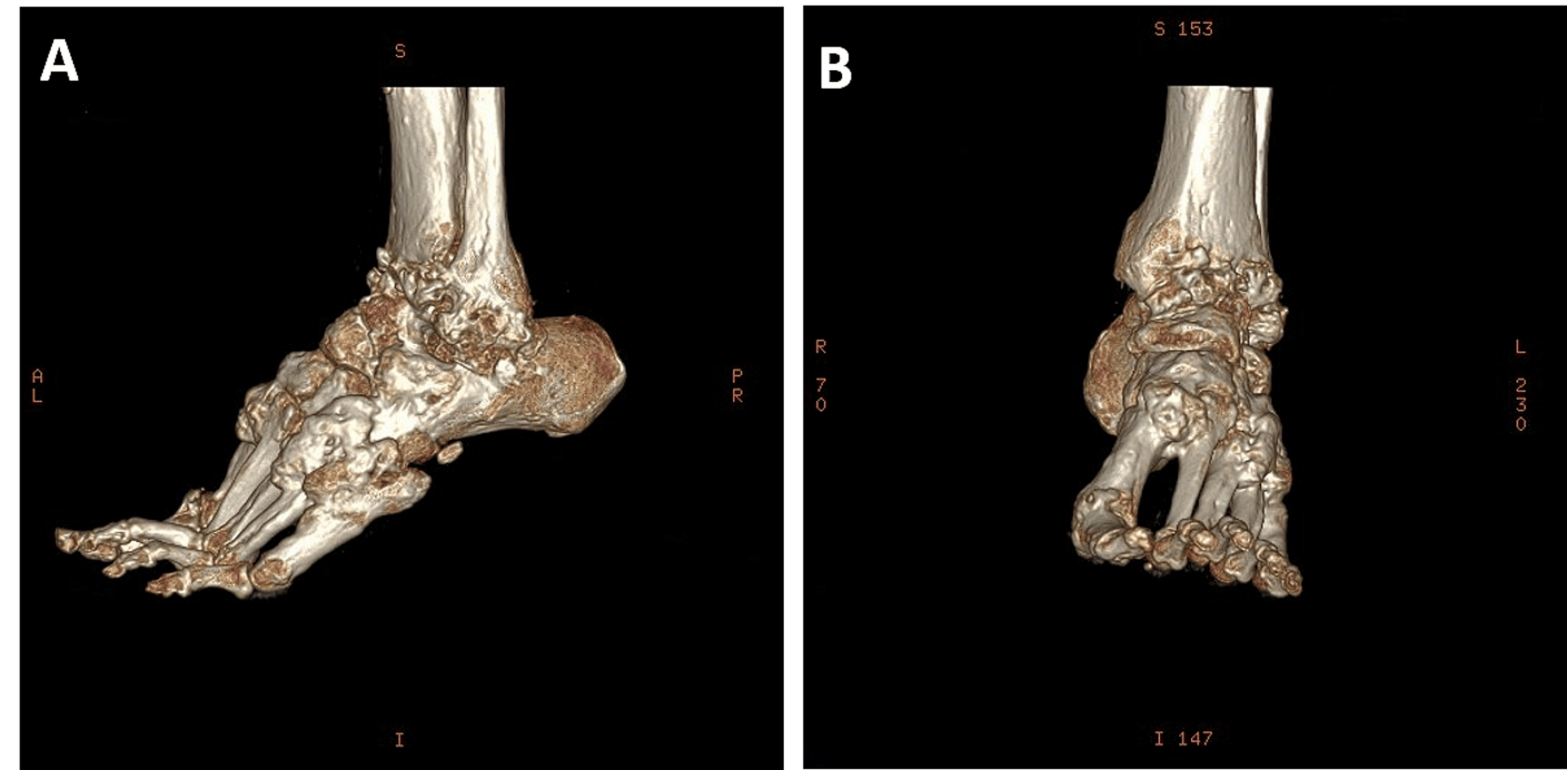
Preoperative three-dimensional computed tomography scan of the left ankle. The images show (A) lateral view and (B) anteroposterior view. Multiple bony spurs are evident around the ankle joint, particularly in the anteromedial to the anterolateral regions.

Plain radiographs revealed multiple anterior spurs with osteoarthritis changes within the ankle joint space. His laboratory test results, including those for tumor markers and inflammatory indices, were all within normal limits. Plain radiographs demonstrated the classic “candle-like flowing wax” appearance, which was suggestive of melorheostosis (Figures 2A, 2B). A subsequent three-dimensional (3D) computed tomography (CT) scan confirmed the diagnosis of left ankle melorheostosis, in addition to identifying a left ankle anterior spur, osteoarthritis, and bony flow across the metatarsal bases (Figures 3A, 3B).

The patient initially received conservative management, including physical therapy, hydrotherapy, and analgesic agents, to provide temporary symptom relief. Owing to the persistent discomfort he experienced, surgical intervention was indicated. A preoperative 3D CT scan was obtained for comprehensive evaluation (Figures 3A, 3B). Subsequently, arthroscopic osteotectomy and ATL with Hoke’s percutaneous triple hemisection were performed.

Surgical procedure

The surgical management of melorheostosis, in this case, involved arthroscopic osteotectomy of ankle joint spurs located on the anterior articular lip, talar dome, and medial and lateral gutters, followed by ATL performed through percutaneous Hoke's triple-cut hemisection. Under spinal anesthesia, the patient was positioned supine, and the left leg was disinfected with a beta-iodine solution and carefully draped. After exsanguination, an anteromedial portal was created to serve as the viewing portal, and an anterolateral portal was created to serve as the working portal. Arthroscopic debridement, osteotectomy of ankle spurs, and synovectomy were performed through the anterolateral portal, with portal switching executed as necessary. Percutaneous ATL was then performed using a Beaver blade no. 64 (Waltham, MA: BVI Medical). After the procedure, the ROM of the patient’s left ankle was manipulated and assessed, and the wound was closed layer by layer after gentle irrigation and placement of a mini-Hemovac drain. A short leg splint was applied posteriorly for external support and protection.

Outcomes

The patient’s initial rehabilitation was performed with an open chain ankle pumping exercise, which was conducted with ankle dorsiflexion and plantarflexion by pulling a towel, and the patient’s foot was not contacted with any object from postoperative day one to two weeks. Partial weight-bearing ambulation with walker assistance was permitted given the patient’s advanced age. At two weeks postoperatively, full weight-bearing ambulation with a walking boot was introduced upon resolution of postoperative pain. Closed-chain ankle exercises, which means that the patient put his foot to a fixed surface while doing ankle dorsiflexion, plantarflexion, and full weight-bearing stance, were initiated at two weeks postoperatively and were continued under the guidance of a physical therapist for two months.

**Figure 4:**
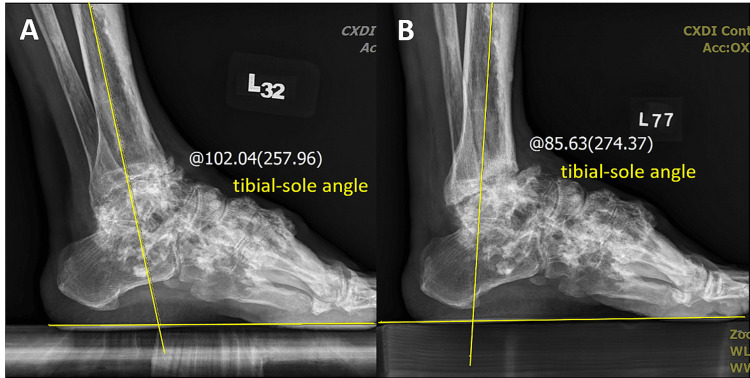
The tibial-sole angle was corrected from preoperative 102.04° to postoperative 85.63° in the lateral view of ankle weight-bearing radiographs. Preoperative (A) and postoperative (B) images.

**Figure 5:**
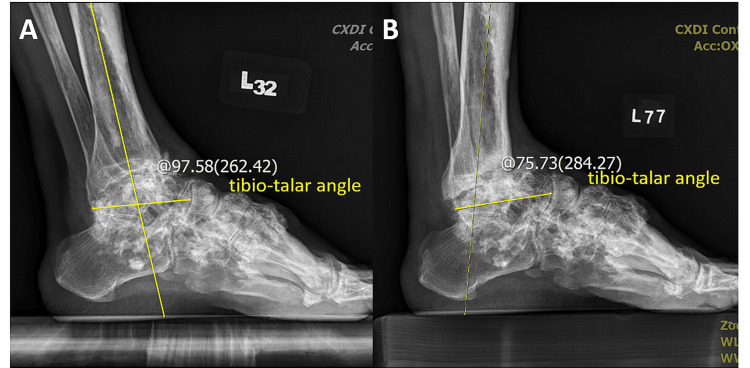
Lateral view of weight-bearing ankle radiographs. Preoperative (A) and postoperative (B) images. The tibio-talar angle was corrected from 97.58° to 75.73°.

**Figure 6:**
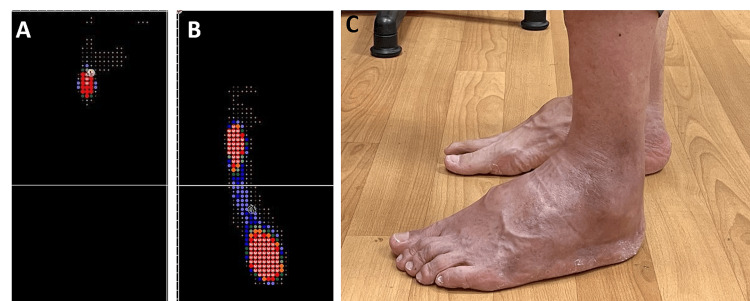
Plantar pressure analysis of the left foot in a static stance. (A) Preoperative view demonstrating weight-bearing primarily on the lateral metatarsal head. (B) Postoperative view demonstrating weight-bearing distribution across the lateral midtarsal and hindfoot. (C) Postoperative lateral view demonstrating improved plantigrade foot position compared with preoperative appearance.

At the six-month follow-up, no significant complication was observed. Plain radiographs revealed improved ankle joint bone alignment compared with preoperative imaging results (Figures 4A, 4B, 5A, 5B). The patient’s left foot tibial-sole angle was corrected from 102.04° to 85.63°, and the tibio-talar angle was corrected from 97.58° to 75.73°. Preoperative plantar pressure analysis revealed a left foot equinovarus pattern with sole weight-bearing limited to the left lateral metatarsal ball (Figures 6A-6C). Postoperatively, the patient exhibited improved ankle range of motion and restored plantigrade and steady gait. He expressed satisfaction with the outcome and was able to resume cycling, a previously enjoyed activity. No local pain was reported (VAS score: 0) over the left ankle and foot during weight-bearing activities or daily life. The patient’s AOFAS Ankle-Hindfoot score improved from preoperative 57 to postoperative 75. The patient currently ambulates independently without assistive devices.

## Discussion

Melorheostosis, first described by Leri and Joanny in 1922, is a rare, chronic bone dysplasia that affects both sexes equally [2]. The onset of this disorder is insidious, and initial manifestations typically involve pain due to subperiosteal bone formation, often leading to restricted ROM and joint deformity [6]. Cutaneous involvement, including hyperpigmentation and scleroderma, as observed in the present case, occurs in approximately 17% of cases (Figures 1A, 1B).

The etiology of melorheostosis remains elusive. Several theories have suggested that melorheostosis may stem from an embryonic mesodermal disorder affecting bones and soft tissues, an infectious process, or vascular insufficiency leading to failure in intramembranous or endochondral ossification [1]. A loss-of-function mutation in LEMD3 (12q12-12q14.3) may result in the encoding of a nonfunctional protein in the inner nuclear membrane, which plays a vital role in the formation of bone morphogenetic proteins and tumor growth factor-beta signaling. However, Hellemans et al. identified no such mutation in isolated and sporadic cases of melorheostosis, indicating a more complex genetic basis [7]. In 1979, Murray and McCredie proposed a monomelic and linear distribution of the disease corresponding to sclerotomes, suggesting that damage to sensory nerve fibers might promote bone scar tissue proliferation [1,8].

Melorheostosis typically manifests during childhood or early adolescence, which is consistent with our patient’s history of symptoms originating in adolescence [9]. The condition progresses gradually, presenting symptoms such as pain, limb swelling, and restricted articular joint ROM. Diagnostic radiographic features include irregular cortical hyperostosis along one side of a long bone, resembling “flowing candle wax.” Routine laboratory findings are generally normal, even with elevated bone-specific alkaline phosphatase activity. Histological examination revealed dense bone without specific cellular abnormalities, rendering it nondiagnostic. Differential diagnoses typically include chronic osteomyelitis, osteopetrosis, osteopoikilosis, osteopathia striata, myositis ossificans, parosteal osteosarcoma, and osteoma. In our patient, the diagnosis was unequivocally established on the basis of clinical presentations and characteristic radiographic and CT findings.

Treatment for melorheostosis primarily focuses on symptom management. Pharmacologic interventions, including nonsteroidal anti-inflammatory drugs, nifedipine, and bisphosphonates, are commonly used for pain management [10,11]. Surgical options, including soft tissue release, hyperostosis excision, and limb lengthening, are reserved for selected patients. Furthermore, surgical debulking of the hyperostotic cortex can alleviate pain and potentially correct deformities. In severe cases with contractures, ischemia, and intractable pain, amputation may be considered as a last resort [12].

**Figure 7:**
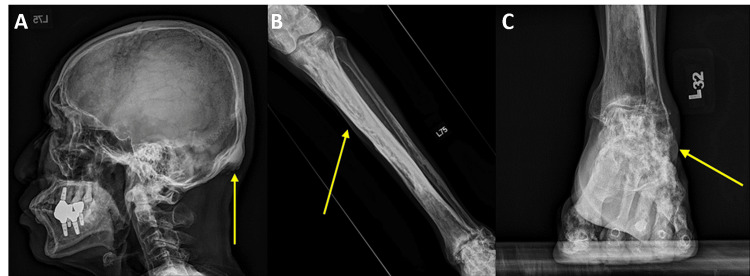
Radiographic findings consistent with melorheostosis. The images show (A) lateral skull view, (B) anteroposterior view of the left tibia, and (C) anteroposterior view of the left ankle. Arrows indicate melorheostosis lesion.

In our patient, melorheostosis affected both the axial and appendicular skeletons, including the skull, tibia, and, in particular, his left foot (Figures 7A-7C). The patient’s chief complaints were peripheral ankle pain, metatarsalgia exacerbated by prolonged standing or walking for more than 20 min, and restricted ROM with a fixed equinus deformity. Considering the critical impact on the patient’s quality of life, surgical intervention was deemed necessary for the management of the fixed equinus deformity.

To minimize soft tissue damage and reduce infection risk, a minimally invasive approach involving arthroscopic osteotectomy and percutaneous ATL was employed. Arthroscopic osteotectomy has demonstrated comparable outcomes to open surgery, with the added benefits of reduced postoperative pain, faster recovery, and shorter rehabilitation time. The posterior heel, a relatively hypovascular region, is susceptible to wound dehiscence and infection. Superficial infection rates for open and percutaneous ATL were reported to be 3.3% and 0%, respectively [13]. Although superficial wound infections can be managed with local debridement and antibiotics, deep wound infections often necessitate repeated wound debridement, local rotational flaps, or free flap transfer to cover the soft tissue defect. The minimally invasive nature of percutaneous Achilles tendon release has contributed to its increasing popularity in recent decades due to reduced wound complication rates. A critical aspect of our surgical procedure involved achieving adequate ankle joint ROM through careful manipulation after bony debulking and soft tissue release. Considering the osteoporotic bone associated with melorheostosis, gentle manipulation is essential to prevent potential iatrogenic fractures. Prompt and aggressive postoperative rehabilitation is also crucial to preventing the recurrence of soft tissue adhesion and ensuring long-lasting protection with casting or splinting.

During the follow-up period, the patient’s outcomes were assessed through plain radiography (Figures 4A, 4B, 5A, 5B), plantar pressure analysis, and gait analysis (Figures 7A-7C). Postoperative radiographs demonstrated correction of the left foot tibial-sole angle (from 102.04° to 85.63°, normal range: 85°-91°), and the tibio-talar angle (from 97.58° to 75.73°, normal range: 64°-72°), indicating improvement in his equinus deformity [14]. Plantar pressure analysis revealed that the foot can bear body weight over the lateral midtarsal and hindfoot portion, indicating enhanced foot pressure distribution during static stance. Furthermore, the patient exhibited a more balanced and comfortable dynamic gait pattern, with restored left ankle ROM, allowing him to resume his previous daily activities. Preoperatively, the patient experienced considerable discomfort due to the ankle ROM being limited to an equinovarus position, which led to difficulty in wearing shoes. This deformity also induced an unsteady gait because he could bear weight only on his right foot. After arthroscopic osteotectomy and ATL, his left ankle was corrected, achieving a plantigrade foot position and a more stable gait pattern.

## Conclusions

Melorheostosis is a benign bone disorder typically managed conservatively with symptomatic treatments; surgical interventions are generally reserved for cases with substantial functional impairment or severe pain in major limb segments (the proximal parts of the extremities). However, the involvement of distal limbs, particularly the hands and feet, often causes mass effect-related signs, including pain, cosmetic concerns, and psychosocial challenges, which may necessitate surgical intervention following conservative treatment failures. Bony debulking and decompression are recommended surgical approaches for distal limb melorheostosis. For ankle involvement with equinus deformity, ATL should be considered. The primary goals of surgical intervention are the amelioration of symptoms, correction of deformities, and restoration of plantigrade foot position and ROM.
